# Evaluation of the Efficacy of Pharmacological Interventions in the Prevention of Acute Mountain Sickness: A Network Meta-Analysis

**DOI:** 10.7759/cureus.111777

**Published:** 2026-06-29

**Authors:** Indrani Sarma, Sukainnya Buragohain, Joonmoni Lahon, Meenakshi Meenu, Satyajit Mohapatra, Dibyajyoti Saikia

**Affiliations:** 1 Pharmacology, All India Institute of Medical Sciences, Guwahati, IND; 2 Pharmacology, All India Institute of Medical Sciences, Bilaspur, IND

**Keywords:** acetazolamide, altitude sickness, budesonide, mountain sickness, theophylline

## Abstract

Acute mountain sickness (AMS) is a common altitude-related condition affecting individuals ascending to high elevations, presenting with headache, nausea, fatigue, and dizziness. Although several pharmacological agents have been proposed for AMS prophylaxis, their comparative effectiveness remains insufficiently established. A systematic review and network meta-analysis were conducted in accordance with Preferred Reporting Items for Systematic Reviews and Meta-Analyses (PRISMA) guidelines. Major electronic databases were searched for randomized controlled trials (RCTs) assessing pharmacological interventions for AMS prophylaxis. Data from 46 eligible studies were extracted using Microsoft Excel (Microsoft Corp., Redmond, WA, USA) and analyzed with R software (R Foundation for Statistical Computing, Vienna, Austria). The primary outcome was AMS incidence. Network geometry was mapped using network plots; treatment effects were calculated as odds ratios (ORs) with 95% confidence intervals (CIs); and interventions were ranked using surface under the cumulative ranking (SUCRA) probabilities. Risk of bias was assessed, and certainty of evidence was determined by CINeMA (Confidence in Network Meta-Analysis). The analysis incorporated 46 studies evaluating multiple pharmacological agents. SUCRA rankings identified inhaled budesonide as the most efficacious agent for AMS prevention, followed by acetazolamide and theophylline. Most studies demonstrated low risk of bias. Acetazolamide exhibited narrower CIs, reflecting a more robust and reliable evidence base. CINeMA showed overall moderate certainty of evidence. Inhaled budesonide had the highest probability of being the most effective pharmacological agent for AMS prevention, with acetazolamide and theophylline also demonstrating substantial efficacy. Acetazolamide remains the best-evidenced option. Additional high-quality RCTs are required to consolidate treatment hierarchies and evaluate agents with limited current data.

## Introduction and background

Acute mountain sickness (AMS) poses a significant health risk to people travelling to high-altitude environments, affecting millions of trekkers, mountaineers, tourists, and occupational workers each year [1]. The condition is characterized by non-specific symptoms-including headache, nausea, vomiting, anorexia, fatigue, dizziness, and sleep disturbance-that typically develop within six to 12 hours of arriving at altitudes above 2,500 m above sea level [1,2]. Reported incidence rates range from approximately 25% at moderate altitudes (2,500-3,500 m) to more than 75% at extreme altitudes exceeding 4,500 m, depending on ascent rate, attained altitude, individual susceptibility, and acclimatization status [3].

The pathophysiology of AMS is complex and multifactorial, involving hypoxia-driven cerebral vasodilation, increased capillary permeability, mild cerebral edema, and disrupted fluid homeostasis [4]. If not promptly recognized and managed, AMS may progress to life-threatening high-altitude cerebral edema (HACE) or high-altitude pulmonary edema (HAPE), both of which carry substantial morbidity and mortality [5]. The growing popularity of high-altitude destinations has heightened the clinical and public health importance of evidence-based AMS prevention [6].

A range of pharmacological agents has been investigated for AMS prophylaxis. Acetazolamide, a carbonic anhydrase inhibitor, has the most extensive research background and is widely recommended as a first-line prophylactic agent [7]. Other approaches-including corticosteroids (dexamethasone and inhaled budesonide), phosphodiesterase inhibitors (theophylline, tadalafil), non-steroidal anti-inflammatory drugs (ibuprofen), and miscellaneous agents-have been evaluated in clinical trials with varying degrees of supporting evidence [8,9]. Direct head-to-head comparisons among all available agents are, however, limited, leaving the relative efficacy of these treatments incompletely characterized.

Network meta-analysis (NMA) is an advanced statistical technique that simultaneously compares multiple interventions by integrating direct evidence from head-to-head trials with indirect evidence via shared comparators [10]. NMA facilitates comprehensive treatment ranking even when direct comparative data are absent, thereby generating clinically applicable guidance for treatment selection [11]. The approach has achieved broad acceptance as a tool for evidence synthesis when multiple treatment options exist but direct comparison data are sparse [12].

The clinical significance of AMS extends beyond personal discomfort to encompass economic, operational, and safety consequences. In well-frequented trekking regions such as the Everest Base Camp route, AMS constitutes a major cause of medical evacuations and rescue operations, with substantial costs and risks for both patients and rescue teams [13]. Military and occupational activities at high altitude are similarly affected by AMS-related morbidity and impaired performance [14]. The potential for AMS to evolve into HACE or HAPE underscores the importance of robust prevention strategies [15].

Individual risk varies widely; established risk factors include rapid ascent, higher target altitude, a prior history of AMS, younger age, and possibly genetic determinants of hypoxic ventilatory response [16]. Even individuals with a history of uneventful high-altitude exposures remain at risk during subsequent ascents, highlighting the ongoing relevance of pharmacological prophylaxis alongside gradual acclimatization [17]. Standardized diagnostic tools such as the Lake Louise Score facilitate AMS research and clinical evaluation [18].

The primary aim of this NMA was to systematically compare the efficacy of all available pharmacological interventions for AMS prevention. Secondary objectives were to rank treatments by probability of superiority, evaluate evidence consistency and quality, and identify research gaps. The findings are intended to provide clinicians, travelers, and policymakers with comprehensive, evidence-based recommendations.

## Review

Methods

Search Strategy and Study Selection

**Figure 1:**
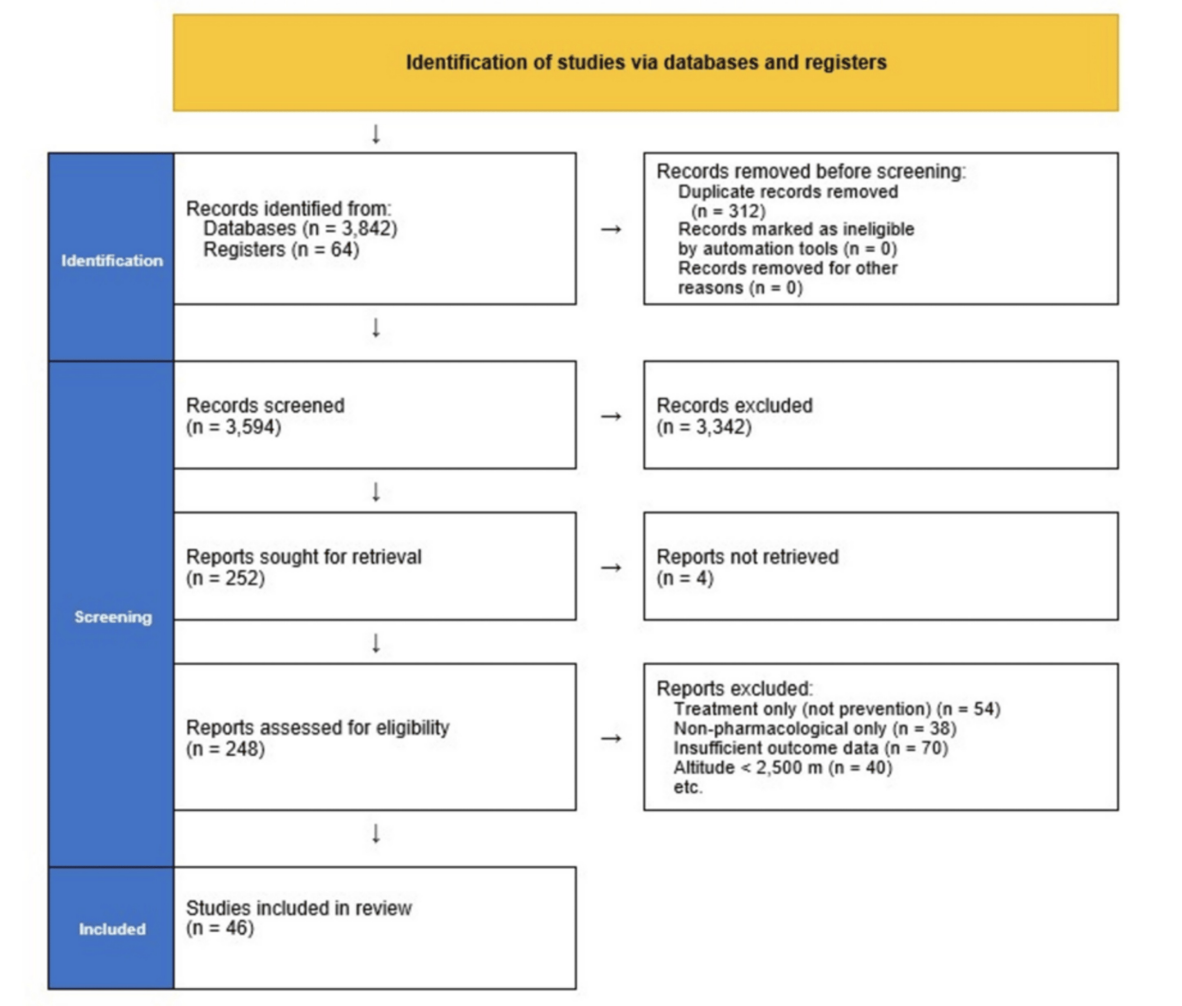
PRISMA flowchart showing the selection process of studies PRISMA: Preferred Reporting Items for Systematic Reviews and Meta-Analyses

This systematic review and NMA was conducted in accordance with the Preferred Reporting Items for Systematic Reviews and Meta-Analyses (PRISMA) 2020 guidelines (Figure 1) [19].

A comprehensive literature search was performed across PubMed/MEDLINE, Embase, the Cochrane Central Register of Controlled Trials (CENTRAL), Web of Science, and Scopus, with no language or date restrictions applied. Search terms combined Medical Subject Headings (MeSH) and free-text keywords relating to AMS, altitude sickness, high-altitude illness, and all pharmacological agents. Terms for study design (randomized controlled trial, clinical trial) and outcomes (prevention, prophylaxis, and incidence) were also incorporated. Full search terms and baseline characteristics of included studies are included in Appendices A-I. The literature search was conducted from database inception to May 25, 2026.

Studies were eligible for inclusion if they were randomized controlled trials (RCTs) involving participants ascending to high altitude, defined as ≥2,500 m, and evaluated at least one pharmacological intervention (acetazolamide, intranasal acetazolamide formulation AR36, budesonide, inhaled budesonide, budesonide-formoterol, dexamethasone, theophylline, tadalafil, sildenafil, ibuprofen, nifedipine, procaterol, inhaled salbutamol, spironolactone, magnesium citrate, carbasalate, sumatriptan, antioxidants, intravenous iron, erythropoietin, trimetazidine, montelukast, zileuton, and *Ginkgo biloba*) for the prevention of AMS. Studies were eligible if they assessed AMS incidence or AMS severity using a validated or clearly defined diagnostic criterion. Eligibility was not restricted to trials in which AMS was the primary endpoint, because several altitude-exposure RCTs primarily investigated pulmonary hemodynamics, HAPE prevention, exercise capacity, cerebral blood flow, or physiological responses, but also prospectively collected AMS outcomes. Such studies were included when the AMS data were extractable and clinically interpretable. Eligible studies were required to include a control group, either placebo or an active comparator, and to report AMS incidence or severity using validated diagnostic criteria, such as the Lake Louise Score. Studies were excluded if they focused exclusively on the treatment of established AMS rather than prophylaxis, evaluated only non-pharmacological interventions, or did not provide sufficient outcome data for analysis. Two independent reviewers screened titles and abstracts, followed by full-text review of potentially eligible studies; discrepancies were resolved by consensus or third-reviewer adjudication. The selection process was documented in a PRISMA flow diagram.

Data Extraction

Data were extracted using a standardized form in Microsoft Excel (Microsoft Corp., Redmond, WA, USA) by two independent reviewers, with discrepancies resolved by reference to the original publication. Extracted variables included study characteristics, participant characteristics, intervention details, control group details, outcome measures, and safety data.

When a study reported outcomes at multiple time points or altitudes, data from the primary time point or the highest altitude were used. Where multiple doses of the same agent were evaluated, each dose group was treated as a distinct network node. For multi-arm trials, all relevant pairwise comparisons were extracted.

Statistical Analysis

NMA was performed in R (version 4.4.0 (Puppy Cup); R Foundation for Statistical Computing, Vienna, Austria) using the netmeta and meta packages. The primary outcome, AMS incidence, was treated as a dichotomous variable, and treatment effects were expressed as odds ratios (ORs) with 95% confidence intervals (CIs); OR < 1 indicated reduced AMS risk relative to the comparator. Network geometry was visualized using network plots; node size reflected the number of participants per intervention, and line thickness reflected the number of studies per direct comparison.

A random-effects NMA model was employed to account for anticipated heterogeneity due to differences in study populations, altitude profiles, ascent rates, and methodologies [20]. Treatment effects were estimated by combining direct and indirect evidence using a frequentist graph-theoretic approach [21]. Forest plots were generated to display pairwise comparison estimates alongside direct evidence where available.

Quality Assessment

Consistency between direct and indirect evidence was evaluated using the node-splitting method; significant inconsistency (p < 0.05) would indicate potential violation of the transitivity assumption [22]. Treatment rankings were derived using surface under the cumulative ranking (SUCRA) probabilities, which represent the likelihood that each treatment ranks among the best options (0% = certainty of worst; 100% = certainty of best) [23]. Publication bias was assessed using comparison-adjusted funnel plots [24].

Certainty of evidence and risk of bias: Risk of bias and certainty of evidence were determined using CINeMA (Confidence in Network Meta-Analysis) [25].

Results

Study Characteristics

The systematic search identified 46 RCTs meeting all inclusion criteria [26-71]. These studies collectively enrolled several thousand participants across diverse geographical settings as well as hypobaric chamber facilities (Appendix I). Sample sizes ranged from small pilot studies (<30 participants) to larger multi-center trials with several hundred participants. Studies were published over several decades, reflecting sustained research interest in pharmacological AMS prophylaxis.

Final altitudes ranged from approximately 2,500 m to above 5,000 m. Ascent protocols varied from rapid ascent designs to gradual ascent itineraries, more representative of typical trekking. This heterogeneity in study design reflects the diverse clinical contexts in which AMS prevention is relevant.

Network Geometry

**Figure 2:**
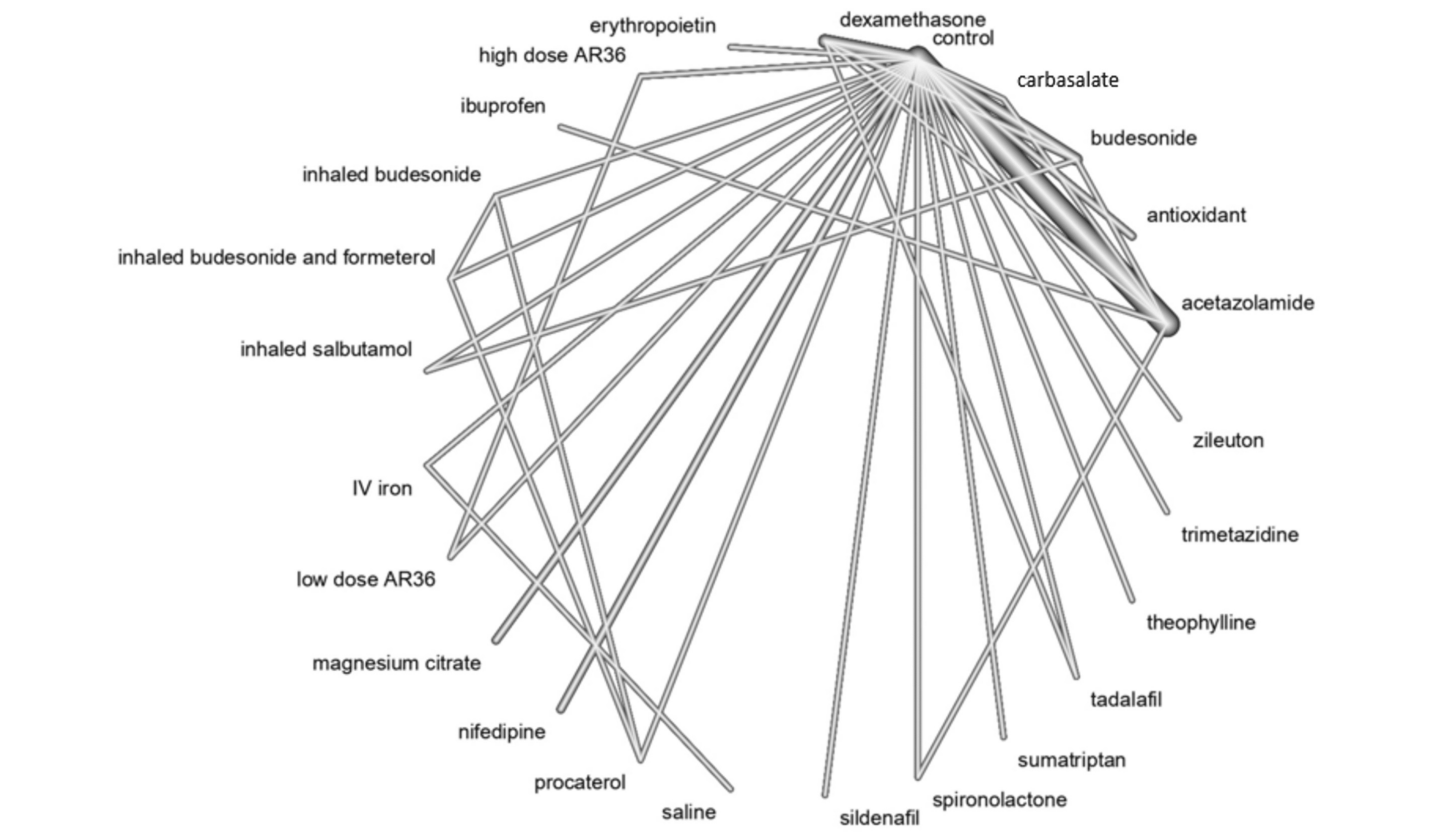
Network geometry of pharmacological interventions included in the network meta-analysis The network plot displays the direct comparisons among pharmacological interventions evaluated for prophylaxis of acute mountain sickness. Each node represents an intervention, and each connecting line represents at least one direct head-to-head comparison between two interventions in the included randomized controlled trials. The thickness of the connecting lines reflects the amount of direct evidence contributing to that comparison, with thicker lines indicating comparisons informed by a larger number of studies. The control group is centrally connected to several active interventions, indicating that many comparisons were placebo/control-based rather than true head-to-head comparisons between active treatments. AR36: a proprietary intranasal acetazolamide formulation; IV: intravenous Figure generated using R software version 4.4.0 (Puppy Cup; R Foundation for Statistical Computing, Vienna, Austria)

The network plot revealed a well-connected structure with multiple pharmacological agents represented as nodes (Figure 2).

Node size was proportional to the number of participants per treatment; line thickness was proportional to the number of direct comparison studies. Acetazolamide constituted the central hub, having direct comparisons with placebo and numerous alternative agents, reflecting its longstanding role as the reference standard. Placebo was also a common comparator.

Inhaled budesonide, dexamethasone, theophylline, ibuprofen, and other agents formed additional nodes with varying degrees of direct and indirect evidence. The network structure permitted estimation of treatment effects for all pairwise comparisons, including those lacking direct head-to-head data.

Comparative Efficacy

**Figure 3:**
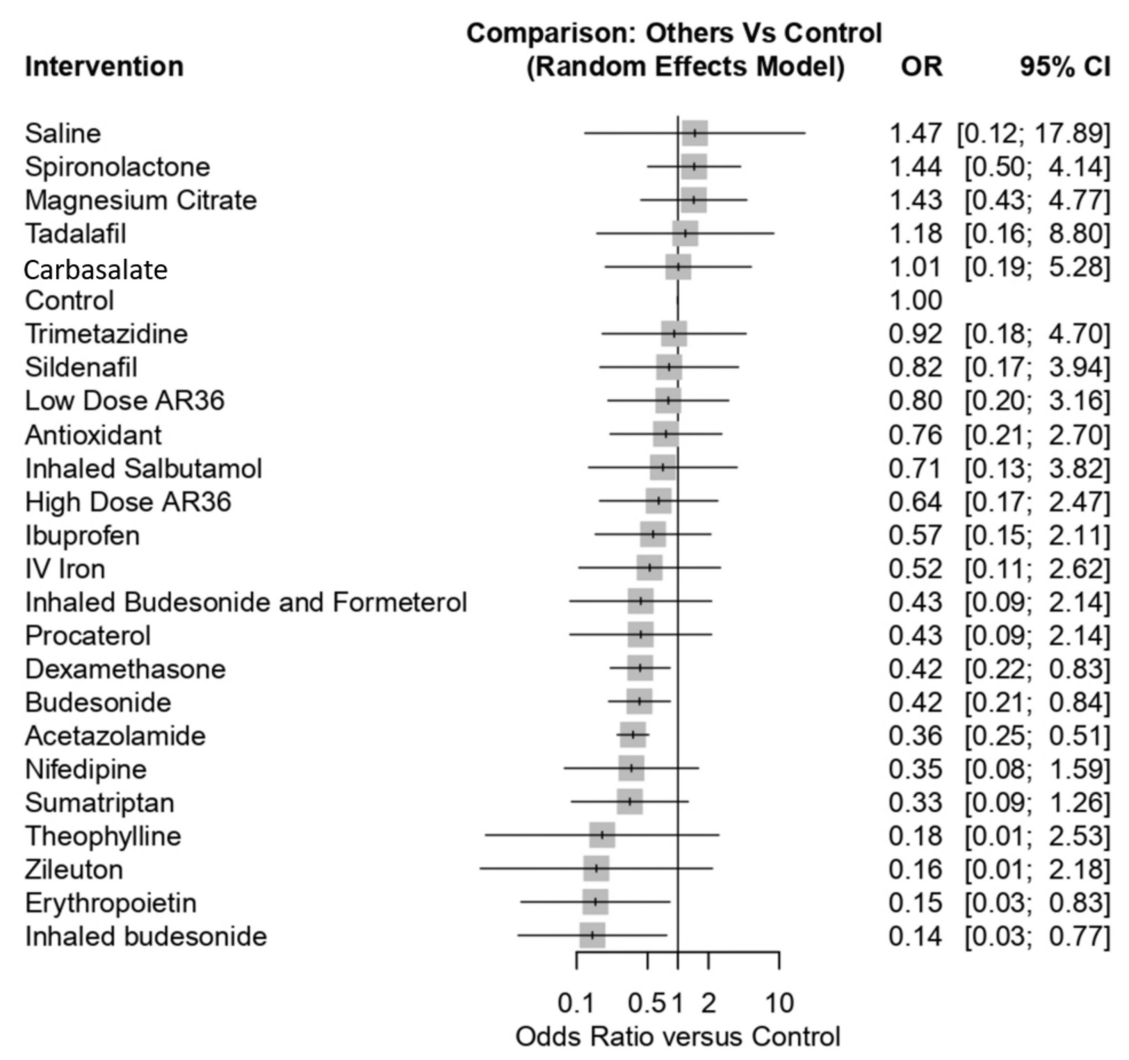
Forest plot comparing pharmacological interventions with control using a random-effects model Forest plot displaying the network meta-analysis results of pharmacological interventions for the prevention of acute mountain sickness, comparing all treatments versus control using a random-effects model. Odds ratios (ORs) with 95% confidence intervals (CIs) are presented here. Treatments are listed in descending order of OR. Saline [53]; spironolactone [36]; magnesium citrate [29,43]; tadalafil [66]; carbasalate [32]; control [26-29,32,35-71]; trimetazidine [62]; sildenafil [68]; low-dose AR36 [27]; antioxidant [44,49]; inhaled salbutamol [71]; high-dose AR36 [27]; ibuprofen [46]; IV iron [48,53]; procaterol [37]; inhaled budesonide and formoterol [37]; dexamethasone [28,35,42,64,66]; budesonide [26,28,64,71]; acetazolamide [26,30-36,39-41,45-47,51,57-61,63,67,69,70]; nifedipine [54,65]; sumatriptan [38]; theophylline [52]; zileuton [50]; erythropoietin [56]; inhaled budesonide [37]. AR36: a proprietary intranasal acetazolamide formulation; IV: intravenous Figure generated using R software version 4.4.0 (Puppy Cup; R Foundation for Statistical Computing, Vienna, Austria)

Forest plots displayed OR estimates with 95% CIs for key pairwise comparisons, combining direct and indirect evidence (Figure 3).

Few interventions showed statistically significant protective effects relative to placebo. The magnitude of benefit varied across agents. Direct and network estimates showed good concordance where both were available, supporting the validity of the NMA. Acetazolamide demonstrated consistent efficacy across multiple comparisons with notably narrow CIs, reflecting the substantial body of evidence supporting its use and providing clinicians with reliable effect estimates.

Treatment Rankings

**Figure 4:**
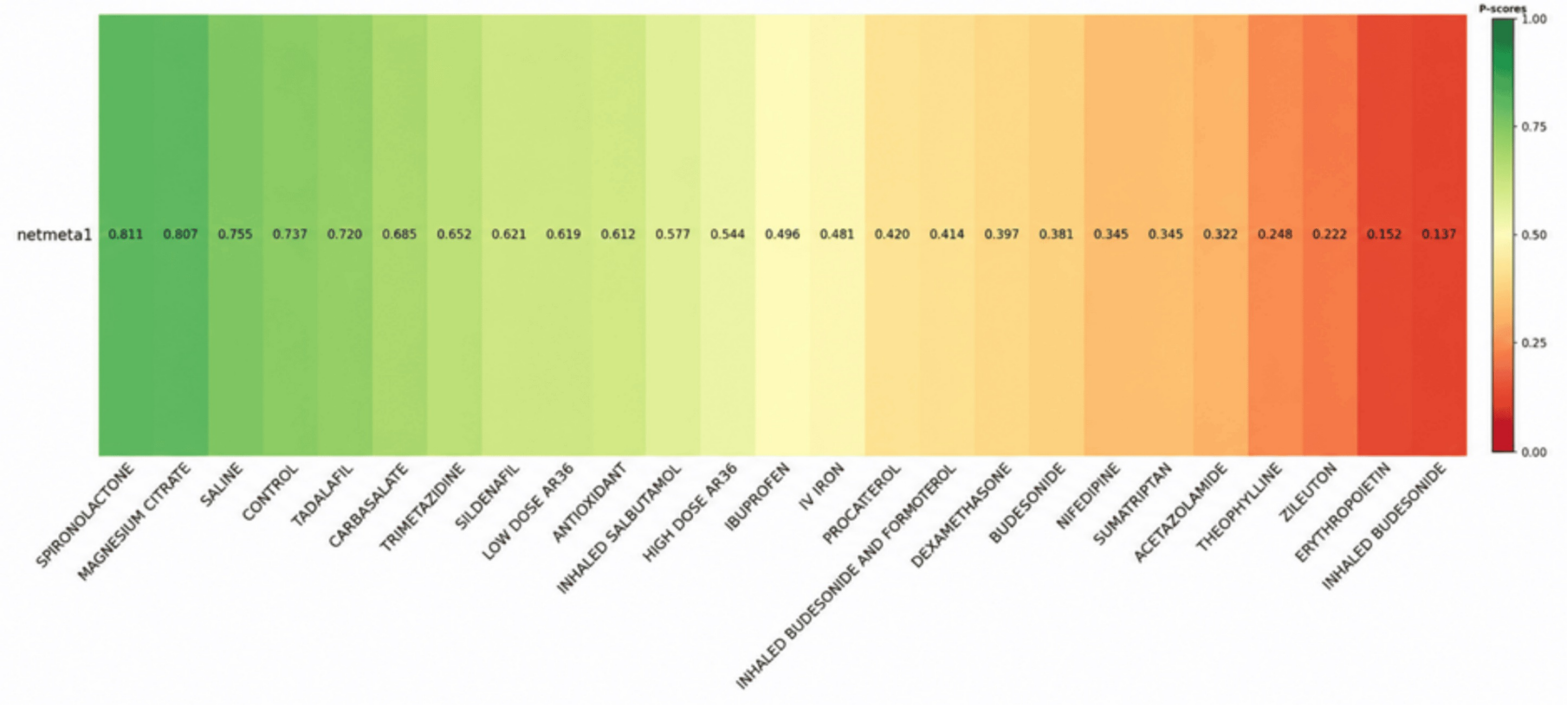
Treatment ranking heat plot based on P-scores from the network meta-analysis The figure presents the relative ranking of pharmacological interventions using P-scores derived from the network meta-analysis. P-scores range from 0 to 1, with higher values indicating a more favorable ranking, depending on the direction in which the outcome was coded. The color gradient represents the magnitude of the P-score, with green indicating higher-ranked interventions and orange/red indicating lower-ranked interventions. In this analysis, spironolactone, magnesium citrate, saline, control, and tadalafil had the highest P-scores, whereas zileuton, erythropoietin, and inhaled budesonide had the lowest P-scores. Here, the outcome assessed was the incidence of acute mountain sickness. Incidence was found to be lowest with inhaled budesonide, indicating prevention was highest for this intervention. AR36: a proprietary intranasal acetazolamide formulation; IV: intravenous Figure generated using R software version 4.4.0 (Puppy Cup; R Foundation for Statistical Computing, Vienna, Austria)

SUCRA analysis produced a comprehensive ranking of all evaluated interventions (Figure 4).

Inhaled budesonide achieved the highest SUCRA value, indicating the greatest probability of being the most effective agent for AMS prevention. Acetazolamide ranked highly, with the advantage of substantially narrower CIs reflecting a more robust evidence base. Theophylline also demonstrated favorable SUCRA rankings, positioning it as a viable alternative. Other agents showed variable SUCRA values reflecting their differing evidence bases. Rankograms and SUCRA plots illustrated that although inhaled budesonide had the highest probability of being the best treatment, there was overlap among probability distributions for top-ranked agents, indicating residual uncertainty in relative rankings.

Risk of Bias

**Figure 5:**
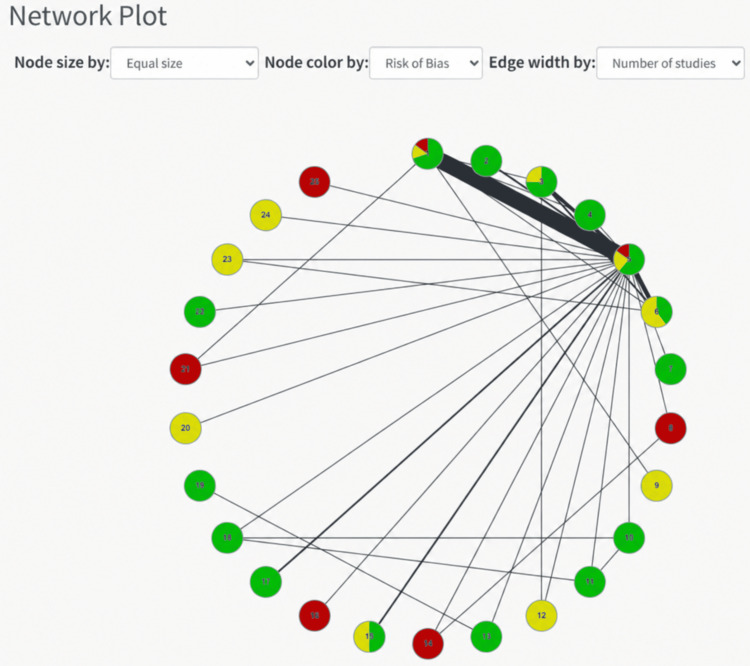
Network plot showing risk-of-bias distribution across treatment comparisons in CINeMA Network plot of included interventions. Each node represents one intervention, labelled according to the treatment code used in the network meta-analysis dataset. Node color indicates the risk-of-bias profile of studies contributing to that intervention, with green, yellow, and red representing lower, some concerns/moderate, and high risk of bias, respectively. Edge width is proportional to the number of direct comparative studies between interventions. The treatment code is as follows: 1: acetazolamide; 2: antioxidant; 3: budesonide; 4: carbasalate; 5: control; 6: dexamethasone; 7: erythropoietin; 8: high-dose AR36; 9: ibuprofen; 10: inhaled budesonide; 11: inhaled budesonide and formoterol; 12: inhaled salbutamol; 13: IV iron; 14: low-dose AR36; 15: magnesium citrate; 16: nifedipine; 17: procaterol; 18: saline; 19: sildenafil; 20: spironolactone; 21: sumatriptan; 22: tadalafil; 23: theophylline; 24: trimetazidine; 25: zileuton AR36: a proprietary intranasal acetazolamide formulation; IV: intravenous Figure generated using CINeMA (Confidence in Network Meta-Analysis) [25]

RoB2 assessment showed that the majority of included studies had a low risk of bias. Adequate randomization and allocation concealment minimized bias from the randomization process. Use of placebo controls and double-blind designs reduced performance and detection bias. Standardized outcome assessment using the Lake Louise Score further controlled measurement bias (Figure 5).

Some studies raised concerns regarding missing outcome data or selective reporting; however, these were infrequent and did not substantially compromise overall evidence quality. No study was judged to have a high risk of bias across multiple domains, warranting exclusion. Sensitivity analyses excluding studies with some concerns yielded results consistent with the primary analysis.

CINeMA

Confidence in the mixed-evidence estimates assessed using the CINeMA framework was generally limited (Table 1).

**Table 1:** CINeMA assessment of confidence in mixed evidence from the network meta-analysis The table presents the Confidence in Network Meta-Analysis (CINeMA) evaluation for each treatment comparison across six domains: within-study bias, reporting bias, indirectness, imprecision, heterogeneity, and incoherence; ratings were classified as high, moderate, low, or very low. Imprecision reflects uncertainty in the pooled effect estimate due to wide confidence intervals or small sample sizes. Heterogeneity reflects variability in effect estimates across direct studies contributing to a comparison. Incoherence reflects inconsistency between direct and indirect evidence within the network. Numbers in square brackets in the Number of studies column denote the reference numbers of the individual studies contributing direct evidence to each pairwise comparison.

Sl. No.	Code	Comparison	Number of studies (references)	Imprecision	Heterogeneity	Incoherence	Confidence rating	Reason(s) for downgrading
1	01:03	Acetazolamide vs. budesonide	1 [26]	Major concerns	No concerns	No concerns	Moderate	Imprecision
2	01:04	Acetazolamide vs. carbasalate	1 [32]	Major concerns	No concerns	No concerns	Moderate	Imprecision
3	01:05	Acetazolamide vs. control	18 [26,32,35,36,39,40,45,47,51,57-61,63,67,69,70]	No concerns	Major concerns	No concerns	Moderate	Heterogeneity
4	01:06	Acetazolamide vs. dexamethasone	1 [35]	Major concerns	No concerns	No concerns	Moderate	Imprecision
5	01:09	Acetazolamide vs. ibuprofen	1 [46]	Major concerns	No concerns	Major concerns	Low	Imprecision, incoherence
6	01:21	Acetazolamide vs. spironolactone	1 [36]	No concerns	Major concerns	No concerns	Moderate	Heterogeneity
7	02:05	Antioxidant vs. control	2 [44,49]	Major concerns	No concerns	Major concerns	Low	Imprecision, incoherence
8	03:05	Budesonide vs. control	4 [26,28,64,71]	No concerns	Major concerns	No concerns	Moderate	Heterogeneity
9	03:06	Budesonide vs. dexamethasone	2 [28,64]	Major concerns	No concerns	No concerns	Moderate	Imprecision
10	04:05	Carbasalet vs. control	1 [32]	Major concerns	No concerns	No concerns	Moderate	Imprecision
11	05:06	Control vs. dexamethasone	5 [28,35,42,64,66]	No concerns	Major concerns	Major concerns	Low	Heterogeneity, incoherence
12	05:07	Control vs. erythropoietin	1 [56]	No concerns	Major concerns	Major concerns	Low	Heterogeneity, incoherence
13	05:08	Control vs. high-dose AR36	1 [27]	Major concerns	No concerns	Major concerns	Low	Imprecision, incoherence
14	10:05	Inhaled budesonide vs. control	1 [37]	No concerns	Major concerns	Major concerns	Low	Heterogeneity, incoherence
15	10:11	Inhaled budesonide vs. inhaled budesonide + formoterol	1 [37]	Major concerns	No concerns	Major concerns	Low	Imprecision, incoherence
16	10:18	Inhaled budesonide vs. procaterol	1 [37]	Major concerns	No concerns	Major concerns	Low	Imprecision, incoherence
17	11:05	Inhaled budesonide + formoterol vs. control	1 [37]	Major concerns	No concerns	Major concerns	Low	Imprecision, incoherence
18	11:18	Inhaled budesonide + formoterol vs. procaterol	1 [37]	Major concerns	No concerns	Major concerns	Low	Imprecision, incoherence
19	12:03	Inhaled salbutamol vs. budesonide	1 [71]	Major concerns	No concerns	No concerns	Moderate	Imprecision
20	12:05	Inhaled salbutamol vs. control	1 [71]	Major concerns	No concerns	No concerns	Moderate	Imprecision
21	13:05	IV iron vs. control	1 [48]	Major concerns	No concerns	Major concerns	Low	Imprecision, incoherence
22	13:19	IV iron vs. saline	1 [53]	Major concerns	No concerns	Major concerns	Low	Imprecision, incoherence
23	14:05	Low-dose AR36 vs. control	1 [27]	Major concerns	No concerns	Major concerns	Low	Imprecision, incoherence
24	14:08	Low-dose AR36 vs. high-dose AR36	1 [27]	Major concerns	No concerns	Major concerns	Low	Imprecision, incoherence
25	15:05	Magnesium citrate vs. control	2 [29,43]	Major concerns	No concerns	Major concerns	Low	Imprecision, incoherence
26	16:05	Montelukast vs. control	1 [55]	Major concerns	No concerns	Major concerns	Low	Imprecision, incoherence
27	17:05	Nifedipine vs. control	2 [54,65]	Major concerns	No concerns	Major concerns	Low	Imprecision, incoherence
28	18:05	Procaterol vs. control	1 [37]	Major concerns	No concerns	Major concerns	Low	Imprecision, incoherence
29	19:05	Sildenafil vs. control	1 [68]	Major concerns	No concerns	Major concerns	Low	Imprecision, incoherence
30	20:05	Spironolactone vs. control	1 [36]	Major concerns	No concerns	No concerns	Moderate	Imprecision
31	21:05	Sumatriptan vs. control	1 [38]	Major concerns	No concerns	Major concerns	Low	Imprecision, incoherence
32	22:05	Tadalafil vs. control	1 [66]	Major concerns	No concerns	No concerns	Moderate	Imprecision
33	22:06	Tadalafil vs. dexamethasone	1 [66]	Major concerns	No concerns	No concerns	Moderate	Imprecision
34	23:05	Theophylline vs. control	1 [52]	Major concerns	No concerns	Major concerns	Low	Imprecision, incoherence
35	24:05	Trimetazidine vs. control	1 [62]	Major concerns	No concerns	Major concerns	Low	Imprecision, incoherence
36	25:05	Zileuton vs. control	1 [50]	Major concerns	No concerns	Major concerns	Low	Imprecision, incoherence

Across 36 mixed-evidence comparisons, none were rated as high confidence; 13 comparisons were judged to have moderate confidence, and 23 low confidence [25]. The most frequent reason for downgrading was major concerns related to imprecision together with incoherence, followed by imprecision alone, whereas a smaller number of estimates were downgraded because of heterogeneity alone or heterogeneity together with incoherence. Most comparisons were informed by sparse evidence, with 29 of 36 comparisons based on a single study [26,27,32,35-38,46,48,50,52,53,55,56,62,66,68,71] and 33 of 36 based on two or fewer studies [26-29,32,35-38,43,44,46,48-50,52-56,62,64-66,68,71]. Comparisons rated as moderate confidence included budesonide versus high-dose AR36 [27], control versus high-dose AR36 [27], control versus dexamethasone [28,35,42,64,66], and several other pairwise contrasts in which downgrading was driven by a single domain only [26,28,32,35,36,39,40,45,47,51,57-61,63,64,66,67,69-71]. In contrast, low-confidence ratings were predominantly observed for comparisons involving major concerns about both imprecision and incoherence [27,37,38,44,46,48-50,52,53-56,62,65,68], indicating substantial uncertainty in the corresponding network estimates. Overall, these findings suggest that although the network permitted estimation of multiple treatment contrasts, the certainty of evidence for most mixed comparisons was low to moderate and should be interpreted cautiously.

Discussion

Principal Findings

This NMA of 46 RCTs provides the most comprehensive synthesis to date of pharmacological interventions for AMS prevention [26-71]. Inhaled budesonide demonstrated the highest SUCRA probability of being the most effective intervention, followed by acetazolamide and theophylline. These findings indicate that several pharmacological options with demonstrated efficacy are available, allowing individualized selection based on patient characteristics, contraindications, side-effect profiles, and practical considerations.

The identification of inhaled budesonide as the top-ranked agent carries potential clinical significance. Its efficacy may relate to potent glucocorticoid-mediated anti-inflammatory and anti-edema effects that directly counter key AMS pathophysiological mechanisms. The inhaled route may optimize the benefit-risk balance by maximizing pulmonary exposure while limiting systemic effects.

Acetazolamide's strong performance, extensive evidence base, and narrow CIs confirm its continued role as a reliable first-line prophylactic agent. The narrower CIs for acetazolamide compared with most other interventions reflect greater precision derived from a larger body of evidence, providing greater clinical certainty. Theophylline's favorable ranking is noteworthy given its distinct mechanism of action and may be particularly relevant for individuals with contraindications to acetazolamide or corticosteroids.

Pharmacological Mechanisms

The efficacy of the highest-ranked agents is explicable through their distinct but complementary mechanisms. Acetazolamide inhibits carbonic anhydrase, promoting renal bicarbonate excretion, inducing compensatory metabolic acidosis, stimulating ventilation, and improving oxygenation, effectively accelerating natural acclimatization [72]. It also reduces cerebrospinal fluid production, potentially attenuating altitude-associated rises in intracranial pressure [73].

Inhaled budesonide appears promising but currently lacks sufficient high-certainty evidence to replace acetazolamide as the preferred first-line prophylactic agent [74]. Potential mechanisms may include stabilization of vascular permeability and attenuation of hypoxia-induced inflammatory responses. The inhaled route achieves high pulmonary drug concentrations while minimizing systemic exposure [1]. Theophylline stimulates respiration and enhances diaphragmatic contractility through phosphodiesterase inhibition and adenosine receptor antagonism, improving ventilatory response to hypoxia [30]; adenosine receptor blockade may additionally reduce cerebral blood flow and attenuate hypoxia-induced cerebral vasodilation, contributing to altitude headache [28].

Clinical Implications

These findings have several practical implications. Inhaled budesonide warrants consideration as a first-line option, particularly for individuals at elevated AMS risk or with contraindications to acetazolamide, though availability, cost, and the requirement for an inhaler device may limit feasibility in remote settings. Acetazolamide remains suitable for most individuals given its oral administration, established dosing, decades of clinical experience, and well-characterized safety profile; clinicians should, however, be aware of contraindications, including sulfonamide allergy and severe renal impairment [75]. Theophylline is a valuable alternative, but its narrow therapeutic index and potential drug interactions necessitate careful dosing and, in some settings, serum level monitoring.

The availability of multiple effective agents with distinct mechanisms supports individualized treatment approaches. Pharmacological prophylaxis should complement, not replace, non-pharmacological strategies including gradual ascent, adequate acclimatization, appropriate hydration, and avoidance of alcohol and sedatives.

Comparison With Existing Literature

These results are broadly consistent with previous systematic reviews. Earlier meta-analyses have established acetazolamide's efficacy for AMS prevention, while meta-analyses specifically addressing inhaled budesonide have reported significant prophylactic benefit, corroborating its high SUCRA ranking here [76,77]. A direct comparative trial by Lipman et al. provided key direct evidence for the budesonide-acetazolamide comparison, which the present NMA extends by simultaneously evaluating multiple agents [26]. The present analysis, incorporating 46 studies, represents the most comprehensive NMA to date and is consistent with prior findings while offering updated rankings.

Limitations

Several limitations should be considered. First, AMS was not uniformly defined across studies; although most used the Lake Louise Score, variations in cut-off values and assessment timing may have introduced heterogeneity in outcome definitions. Second, evidence was sparse for several agents, resulting in wide CIs and greater uncertainty for those treatments; inhaled budesonide, despite its top ranking, has been evaluated in fewer trials than acetazolamide.

Third, publication bias cannot be entirely excluded despite the absence of strong asymmetry on funnel plot inspection; unpublished negative studies may exaggerate treatment effect estimates. Finally, adverse events were not systematically analyzed; future work should incorporate a comprehensive benefit-risk assessment.

Also, several network-specific limitations should be acknowledged. The evidence network was sparse, with many treatment nodes informed by only a single study and relatively few direct head-to-head comparisons between active pharmacological agents. As a result, several estimates relied substantially on indirect evidence and on the assumption of transitivity across trials that differed in altitude exposure, ascent profile, population characteristics, dosing, and AMS definitions. In addition, potential small-study effects or publication bias could not be confidently excluded, particularly for less-studied interventions.

## Conclusions

This NMA synthesized randomized evidence on pharmacological prophylaxis for AMS and suggests that several agents may reduce AMS incidence compared with control. Inhaled budesonide achieved the most favorable ranking in the network analysis; however, this finding should be interpreted cautiously because it was supported by a limited evidence base, sparse network connections, and uncertainty in some comparisons. It should therefore be considered a promising intervention rather than a replacement for acetazolamide in routine practice. Acetazolamide remains the best-established first-line pharmacological option for AMS prophylaxis, supported by the largest and most consistent body of evidence, established clinical use, oral feasibility, and a well-characterized safety profile. Other agents, including dexamethasone, theophylline, phosphodiesterase inhibitors, inhaled beta-agonists, erythropoietin, sumatriptan, and metabolic or anti-inflammatory agents, may have specific theoretical or clinical roles, but the certainty of evidence for many of these interventions remains limited. In particular, theophylline should be used with caution because of its narrow therapeutic index, potential adverse effects, and drug-interaction concerns. Also, inhaled budesonide achieved the highest SUCRA ranking; however, this finding was based on limited evidence with low certainty. Therefore, the apparent superiority of inhaled budesonide should be interpreted cautiously until confirmed by larger RCTs.

Treatment selection for AMS prevention should not be based on efficacy rankings alone. Clinical decisions should also consider tolerability, contraindications, adverse-effect profile, drug interactions, route of administration, availability, cost, and feasibility in remote high-altitude settings. Pharmacological prophylaxis should complement, not replace, established preventive and management strategies such as gradual ascent, adequate acclimatization, early recognition of symptoms, avoidance of alcohol and sedatives, descent when clinically indicated, and oxygen therapy where available. Future trials should use standardized AMS definitions, clearly report dose and route, prospectively assess safety and tolerability, and include adequately powered head-to-head comparisons of promising agents against acetazolamide. Until such evidence is available, treatment rankings from the present NMA should be interpreted as hypothesis-generating and clinically contextual rather than definitive proof of superiority.

## Appendices

Appendix A 

**Table 2:** Search terms for NMA across Scopus NMA: network meta-analysis

#	Concept	Search string
Block A: condition		
1	Condition	TITLE-ABS-KEY("acute mountain sickness" OR "altitude sickness" OR "high altitude illness" OR "altitude illness" OR "mountain sickness" OR "AMS")
Block B: interventions		
2	Drugs	TITLE-ABS-KEY("acetazolamide" OR "dexamethasone" OR "budesonide" OR "ibuprofen" OR "nifedipine" OR "tadalafil" OR "sildenafil" OR "spironolactone" OR "theophylline" OR "sumatriptan" OR "antioxidant*" OR "magnesium" OR "erythropoietin" OR "intravenous iron" OR "trimetazidine" OR "montelukast" OR "zileuton" OR "ginkgo biloba" OR "pharmacological prevention" OR "drug prophylaxis")
Block C: study design		
3	RCT	TITLE-ABS-KEY("randomized controlled trial" OR "randomised controlled trial" OR "RCT" OR "placebo-controlled" OR "double-blind" OR "randomly assigned")
Final	Final query	1 AND 2 AND 3

Appendix B 

**Table 3:** Search terms for network meta-analysis across Embase

#	Concept	Search string (Ovid syntax)
Block A: condition		
1	Disease Emtree	'mountain sickness'/exp OR 'acute mountain sickness'/exp
2	Disease text	('acute mountain sickness':ti,ab,kw OR 'AMS':ti,ab,kw OR 'altitude sickness':ti,ab,kw OR 'high altitude illness':ti,ab,kw OR 'altitude illness':ti,ab,kw OR 'mountain sickness':ti,ab,kw)
3	High altitude	'high altitude'/exp OR 'altitude':ti,ab,kw OR 'hypobaric hypoxia':ti,ab,kw
A	Block A	1 OR 2 OR 3
Block B: interventions		
4	Acetazolamide	'acetazolamide'/exp OR 'acetazolamide':ti,ab,kw OR 'Diamox':ti,ab,kw
5	Dexamethasone	'dexamethasone'/exp OR 'dexamethasone':ti,ab,kw OR 'corticosteroid*':ti,ab,kw
6	Budesonide	'budesonide'/exp OR 'budesonide':ti,ab,kw OR 'inhaled corticosteroid*':ti,ab,kw
7	Ibuprofen	'ibuprofen'/exp OR 'ibuprofen':ti,ab,kw OR 'NSAID*':ti,ab,kw
8	Nifedipine	'nifedipine'/exp OR 'nifedipine':ti,ab,kw OR 'calcium channel blocker*':ti,ab,kw
9	Tadalafil/PDE5i	'tadalafil':ti,ab,kw OR 'sildenafil':ti,ab,kw OR 'phosphodiesterase inhibitor*':ti,ab,kw
10	Others	'spironolactone':ti,ab,kw OR 'theophylline':ti,ab,kw OR 'sumatriptan':ti,ab,kw OR 'antioxidant*':ti,ab,kw OR 'magnesium':ti,ab,kw OR 'erythropoietin':ti,ab,kw OR 'trimetazidine':ti,ab,kw OR 'montelukast':ti,ab,kw OR 'zileuton':ti,ab,kw OR 'ginkgo biloba':ti,ab,kw
B	Block B	4 OR 5 OR 6 OR 7 OR 8 OR 9 OR 10
Block C: study design		
11	RCT Emtree	'randomized controlled trial'/exp
12	RCT text	('randomized controlled trial*':ti,ab,kw OR 'randomised controlled trial*':ti,ab,kw OR 'RCT':ti,ab,kw OR 'placebo-controlled':ti,ab,kw OR 'double-blind*':ti,ab,kw OR 'randomly allocated':ti,ab,kw)
C	Block C	11 OR 12
Final	Final query	A AND B AND C

Appendix C 

**Table 4:** Search terms for network meta-analysis across Cochrane Central Register of Controlled Trials (CENTRAL) RCT: randomized controlled trial

#	Concept	Search string
Block A: condition		
1	MeSH	[mh "Altitude Sickness"] OR [mh "Mountain Sickness, Acute"]
2	Free text	("acute mountain sickness" OR "altitude sickness" OR "AMS" OR "high altitude illness" OR "altitude illness" OR "mountain sickness"):ti,ab,kw
A	Block A	1 OR 2
Block B: interventions		
3	All drugs	(acetazolamide OR dexamethasone OR budesonide OR ibuprofen OR nifedipine OR tadalafil OR sildenafil OR spironolactone OR theophylline OR sumatriptan OR antioxidant* OR magnesium OR erythropoietin OR "intravenous iron" OR trimetazidine OR montelukast OR zileuton OR "ginkgo biloba" OR "pharmacological prevention" OR prophylaxis):ti,ab,kw
B	Block B	3
Final	Final query (CENTRAL is RCT-only-no Block C needed)	A AND B

Appendix D 

**Table 5:** Search terms for the network meta- analysis across PubMed/MEDLINE MeSH: Medical Subject Headings; RCT: randomized controlled trial; NSAIDs: non-steroidal anti-inflammatory drugs; EPO: erythropoietin

#	Concept	Search string
Block A: condition		
1	Disease MeSH	"Altitude Sickness"[MeSH] OR "Mountain Sickness, Acute"[MeSH]
2	Disease text	("acute mountain sickness"[tiab] OR "AMS"[tiab] OR "altitude sickness"[tiab] OR "high altitude illness"[tiab] OR "high altitude disease"[tiab] OR "altitude illness"[tiab] OR "high-altitude sickness"[tiab] OR "mountain sickness"[tiab])
3	High altitude MeSH	"Altitude"[MeSH:NoExp] OR "High Altitude"[MeSH]
4	High altitude text	("high altitude"[tiab] OR "high-altitude"[tiab] OR "hypobaric"[tiab] OR "hypoxia"[tiab] OR "altitude exposure"[tiab])
A	Block A combined	1 OR 2 OR 3 OR 4
Block B: interventions		
5	Acetazolamide	"Acetazolamide"[MeSH] OR "acetazolamide"[tiab] OR "Diamox"[tiab] OR "carbonic anhydrase inhibitor*"[tiab]
6	Dexamethasone	"Dexamethasone"[MeSH] OR "dexamethasone"[tiab] OR "corticosteroid*"[tiab]
7	Budesonide	"budesonide"[tiab] OR "inhaled corticosteroid*"[tiab] OR "inhaled budesonide"[tiab] OR "Pulmicort"[tiab]
8	Ibuprofen/NSAIDs	"Ibuprofen"[MeSH] OR "ibuprofen"[tiab] OR "nonsteroidal anti-inflammatory"[tiab] OR "NSAID*"[tiab]
9	Nifedipine	"Nifedipine"[MeSH] OR "nifedipine"[tiab] OR "calcium channel blocker*"[tiab]
10	Tadalafil/PDE5i	"tadalafil"[tiab] OR "sildenafil"[tiab] OR "phosphodiesterase inhibitor*"[tiab] OR "PDE-5 inhibitor*"[tiab]
11	Spironolactone	"Spironolactone"[MeSH] OR "spironolactone"[tiab]
12	Theophylline	"Theophylline"[MeSH] OR "theophylline"[tiab] OR "aminophylline"[tiab]
13	Sumatriptan	"sumatriptan"[tiab] OR "triptan*"[tiab]
14	Antioxidants	"Antioxidants"[MeSH] OR "antioxidant*"[tiab] OR "vitamin C"[tiab] OR "vitamin E"[tiab]
15	Iron/EPO	"Iron"[MeSH] OR "intravenous iron"[tiab] OR "erythropoietin"[tiab] OR "EPO"[tiab]
16	Magnesium	"magnesium"[tiab] OR "magnesium citrate"[tiab]
17	Others	"trimetazidine"[tiab] OR "montelukast"[tiab] OR "zileuton"[tiab] OR "ginkgo biloba"[tiab] OR "Ginkgo"[MeSH] OR "pharmacological prophylaxis"[tiab] OR "drug prophylaxis"[tiab] OR "prevention"[tiab]
B	Block B combined	5 OR 6 OR 7 OR 8 OR 9 OR 10 OR 11 OR 12 OR 13 OR 14 OR 15 OR 16 OR 17
Block C: study design		
18	RCT MeSH	"Randomized Controlled Trial"[pt] OR "Randomized Controlled Trials as Topic"[MeSH]
19	RCT text	("randomized controlled trial*"[tiab] OR "randomised controlled trial*"[tiab] OR "RCT"[tiab] OR "random allocation"[tiab] OR "randomly assigned"[tiab] OR "placebo-controlled"[tiab] OR "double-blind"[tiab] OR "double blind"[tiab])
C	Block C combined	18 OR 19
Final	Final query	A AND B AND C

Appendix E 

**Table 6:** Search terms for NMA across Web of Science Core Collection NMA: network meta-analysis; RCT: randomized controlled trial

#	Concept	Search string
Block A: condition		
1	Condition	TS=("acute mountain sickness" OR "altitude sickness" OR "high altitude illness" OR "altitude illness" OR "mountain sickness")
Block B: interventions		
2	Drugs	TS=("acetazolamide" OR "dexamethasone" OR "budesonide" OR "ibuprofen" OR "nifedipine" OR "tadalafil" OR "sildenafil" OR "spironolactone" OR "theophylline" OR "sumatriptan" OR "antioxidant*" OR "magnesium" OR "erythropoietin" OR "intravenous iron" OR "trimetazidine" OR "montelukast" OR "zileuton" OR "ginkgo biloba" OR "drug prophylaxis")
Block C: study design		
3	RCT	TS=("randomized controlled trial" OR "randomised controlled trial" OR "RCT" OR "placebo-controlled" OR "double-blind") AND DT=(Article)
Final	Final query	1 AND 2 AND 3

Appendix F 

**Table 7:** Search terms in trial registries

Registry	URL	Search terms used
ClinicalTrials.gov	clinicaltrials.gov	"acute mountain sickness" OR "altitude sickness" OR "AMS prevention" OR "high altitude illness" — Condition/disease field; Intervention field: acetazolamide OR budesonide OR dexamethasone OR ibuprofen
WHO ICTRP	trialsearch.who.int	"acute mountain sickness" OR "altitude illness" — All fields; Recruitment status: All
ISRCTN Registry	isrctn.com	"acute mountain sickness" OR "high altitude sickness" — Free text search
EU Clinical Trials Register	clinicaltrialsregister.eu	"mountain sickness" OR "altitude sickness" — Medical condition field

Appendix G 

**Table 8:** Search terms for gray literature & hand-searching

Source	Approach
Google Scholar	"acute mountain sickness" prevention RCT — First 5 pages; citation tracking of key included studies
Reference lists	Manual screening of reference lists of all included studies and relevant systematic reviews
Wilderness Med. journals	High Altitude Medicine & Biology; Wilderness & Environmental Medicine — Table of contents review (last 5 years)
Expert contact	Contact with corresponding authors of included RCTs to identify unpublished data or ongoing trials

Appendix H 

**Table 9:** Key terms and glossary

Concept	Preferred terms	Synonyms/variants searched
Disease	Acute mountain sickness	AMS, altitude sickness, altitude illness, high altitude illness, high-altitude sickness, mountain sickness
Altitude	High altitude	High-altitude, hypobaric, hypobaric hypoxia, altitude exposure, elevated altitude, >2500 m
Design	Randomized controlled trial	RCT, randomised controlled trial, placebo-controlled trial, double-blind trial, random allocation, randomly assigned
Acetazolamide	Acetazolamide	Diamox, carbonic anhydrase inhibitor, CAI
Budesonide	Inhaled budesonide	Pulmicort, inhaled corticosteroid, ICS, inhaled steroid
Dexamethasone	Dexamethasone	Decadron, corticosteroid, systemic steroid, glucocorticoid
Ibuprofen	Ibuprofen	NSAIDs, non-steroidal anti-inflammatory, COX inhibitor
Nifedipine	Nifedipine	Adalat, calcium channel blocker, CCB
PDE5 inhibitors	Tadalafil, sildenafil	Cialis, Viagra, phosphodiesterase-5 inhibitor

Appendix I 

**Table 10:** Baseline characteristics of included studies A total of 46 studies were included with ~5,500, altitude range: 3,100-5,200 m, years: 1987-2024, interventions: 14 pharmacological agents, countries: 13 RCT: randomized controlled trial; DB: double-blind; PC: placebo-controlled; LLS: Lake Louise Score; AR36: a proprietary intranasal acetazolamide formulation; ACZ: acetazolamide; BUD: budesonide; PL: placebo; DEX: dexamethasone; CBS: carbasalate; ASA: acetylsalicylic acid; RIPC: remote ischemic preconditioning; SAL: salbutamol; SLD: sildenafil; AMS: acute mountain sickness; SB: single-blinded; GB: *Ginkgo biloba*; SPL: spironolactone; PRC: procaterol; F: formoterol; SUM: sumatriptan; XO: cross-over; AO: antioxidant; HAPE: high-altitude pulmonary edema; IBU: ibuprofen; IV: intravenous; ZIL: zileuton; THEO: theophylline; NIF: nifedipine; MTK: montelukast; EPO: erythropoietin; TMZ: trimetazidine; TAD: tadalafil

SL	Ref.	Study	Country	Design	Arms	N	Altitude	AMS tool	Interventions	Dose/regimen	AMS events (n/N)	AMS rate	Notes
1	[26]	Lipman et al., 2018	USA	RCT, DB, PC	3 arms	103	4,928 m (Everest trek)	LLS ≥ 3	BUD/ACZ/PL	BUD 200 µg inh BD; ACZ 250 mg BD	BUD 9/33; ACZ 20/35; PL 13/35	BUD 27%; ACZ 57%; PL 37%	Active comparator design
2	[27]	Schober et al., 2023	USA	RCT, DB, PC	3 arms	106	3,800-4,300 m	LLS 2018 ≥ 3	AR36 low/AR36 high/PL	Low: 50 mg; high: 150 mg	Low 13/33; high 17/38; PL 12/35	Low 39%; high 45%; PL 34%	Novel agent (AR36)
3	[28]	Zheng et al., 2014	China	RCT, DB, PC	3 arms	124	3,800 m (rapid ascent)	LLS ≥ 3	BUD inh/DEX oral/PL	BUD 800 µg/d; DEX 8 mg/d	BUD 32/42; DEX 27/39; PL 17/43	BUD 76%; DEX 69%; PL 40%	Emergency/rapid ascent population
4	[29]	Dumont et al., 2004	Switzerland	RCT, DB, PC	2 arms	61	3,611 m (Monte Rosa)	LLS ≥ 3	Magnesium citrate/PL	Mg 21.4 mmol/d	Mg 6/30; PL 5/31	Mg 20%; PL 16%	Crossover; no significant effect
5	[30]	Drago et al., 2022	Chile	RCT, SB	1 arm vs. hist.	17	3,696 m	LLS ≥ 3	ACZ	ACZ 250 mg BD	ACZ 14/17	ACZ 82%	Single-arm; ventilation protocol
6	[31]	Lipman et al., 2020	USA	RCT, DB, PC	3 arms	106	4,928 m	LLS ≥ 3	ACZ 62.5 mg/125 mg/PL	62.5 mg BD vs. 125 mg BD	ACZ 102/106 (combined arms)	96%	Dose-finding; lowest effective dose
7	[32]	Kayser et al., 2008	Switzerland	RCT, DB, PC	3 arms	75	3,611 m	LLS ≥ 3	CBS/ACZ/PL	CBS 100 mg/d; ACZ 500 mg/d	CBS 3/15; ACZ 20/44; PL 2/16	CBS 20%; ACZ 45%; PL 13%	Carbasalate (ASA analog)
8	[33]	Liu et al., 2024	China	RCT, DB, PC	5 arms	250	4,000 m	LLS ≥ 3	ACZ/RIPC/ACZ + RIPC/sham + ACZ/control	ACZ 250 mg BD	ACZ 43/50	86%	RIPC combination trial; ACZ arm extracted
9	[34]	Chow et al., 2005	USA	RCT, DB, PC	4 arms	116	4,300 m (White Mt)	LLS ≥ 3	ACZ/GB/both/PL	ACZ 250 mg BD; GB 120 mg BD	ACZ 18/24	75%	ACZ arm only extracted
10	[35]	Ellsworth et al., 1987	USA	RCT, DB, PC	3 arms	47	4,392 m (Mt. Rainier)	AMS consensus criteria	ACZ/DEX/PL	ACZ 250 mg BD; DEX 8 mg/d	ACZ 14/15; DEX 16/17; PL 12/15	ACZ 93%; DEX 94%; PL 80%	Early landmark RCT
11	[36]	Basnyat et al., 2011	Nepal	RCT, DB, PC	3 arms	251	4,928 m	LLS ≥ 3	SPL/ACZ/PL	SPL 25 mg TID; ACZ 250 mg BD	SPL 65/92; ACZ 85/95; PL 51/64	SPL 71%; ACZ 89%; PL 80%	SPACE trial; SPL not effective
12	[37]	Chen et al., 2015	China	RCT, DB, PC	4 arms	80	3,658 m	LLS ≥ 3	BUD inh/PRC/BUD + F/PL	BUD 800 µg/d; PRC 50 µg/d	BUD 15/20; PRC 10/20; BUD+F 10/20; PL 6/20	75%; 50%; 50%; 30%	Inhaled drug comparison
13	[38]	Jafarian et al., 2007	Iran	RCT, DB, XO	2 arms	97	3,100 m	LLS ≥ 3	SUM/PL	SUM 50 mg oral	SUM 39/48; PL 29/49	SUM 81%; PL 59%	XO; high-altitude headache
14	[39]	Grissom et al., 1992	USA	RCT, DB, PC	2 arms	12	4,393 m (White Mt.)	AMS criteria	ACZ/PL	ACZ 250 mg BD	ACZ 5/6; PL 0/6	ACZ 83%; PL 0%	Small sample; treatment trial
15	[40]	Hillenbrand et al., 2006	UK	RCT, DB, PC	2 arms	109	4,559 m (Monte Rosa)	LLS ≥ 3	ACZ/PL	ACZ 250 mg BD	ACZ 48/55; PL 48/54	ACZ 87%; PL 89%	Hemostasis focus; AMS secondary outcome
16	[41]	Gertsch et al., 2004	Nepal	RCT, DB, PC	4 arms	487	4,928 m	LLS ≥ 3	ACZ/GB/both/PL	ACZ 250 mg BD	ACZ 104/118	PHAIT 88%	Largest Himalayan trekker RCT (PHAIT)
17	[42]	Furian et al., 2018	Austria/Italy	RCT, DB, PC	2 arms	118	4,559 m	LLS ≥ 3	DEX/PL	DEX 8 mg/d	DEX 47/60; PL 44/58	DEX 78%; PL 76%	Exercise capacity primary outcome
18	[43]	Lysakowski et al., 2004	Switzerland	RCT, DB, PC	2 arms	39	3,611 m	LLS ≥ 3	Magnesium citrate/PL	Mg 21.4 mmol/d	Mg 13/21; PL 15/18	Mg 62%; PL 83%	Cerebral blood flow study
19	[44]	Bailey and Davies, 2001	UK	RCT, DB, PC	2 arms	18	5,200 m (Nepal trek)	LLS ≥ 3	AO/PL	Vit C 1 g + Vit E 400 IU/d	AO 4/9; PL 0/9	AO 44%; PL 0%	Small sample; oxidative stress focus
20	[45]	Berger et al., 2022	Switzerland	RCT, DB, PC	2 arms	13	4,559 m	LLS ≥ 3	ACZ/PL	ACZ 250 mg BD	ACZ 3/7; PL 1/6	ACZ 43%; PL 17%	HAPE-susceptible subjects
21	[46]	Burns et al., 2019	USA	RCT, DB, PC	3 arms	139	3,810-4,300 m	LLS ≥ 3	ACZ/IBU/PL	ACZ 250 mg BD; IBU 600 mg TID	ACZ 23/47; IBU 17/45	ACZ 49%; IBU 38%	Head-to-head ACZ vs. IBU
22	[47]	van Patot et al., 2008	USA	RCT, DB, PC	2 arms	44	4,300 m	LLS ≥ 3	ACZ/PL	ACZ 125 mg BD	ACZ 19/22; PL 11/22	ACZ 86%; PL 50%	Low-dose ACZ; Colorado field study
23	[48]	Ren et al., 2015	China	RCT, DB, PC	2 arms	38	3,700 m	LLS ≥ 3	IV iron/PL	Fe-sucrose 200 mg IV	Fe 12/19; PL 9/19	Fe 63%; PL 47%	Iron supplementation; PLA study
24	[49]	Baillie et al., 2009	UK/Nepal	RCT, DB, PC	2 arms	71	5,200 m	LLS ≥ 3	AO/PL	Vit C 1 g + Vit E 400 IU/d	AO 11/36; PL 12/35	AO 31%; PL 34%	No protective effect
25	[50]	Grissom et al., 2005	USA	RCT, DB, PC	2 arms	18	4,200 m (Denali)	LLS ≥ 3	zileuton/PL	ZIL 600 mg QID	ZIL 8/9; PL 5/9	ZIL 89%; PL 56%	5-LOX inhibitor; small sample
26	[51]	Basnyat et al., 2006	Nepal	RCT, DB, PC	2 arms	176	4,928 m	LLS ≥ 3	ACZ 125 mg/375 mg/PL	125 mg BD vs. 375 mg BD	ACZ 111/126; PL 29/50	ACZ 88%; PL 58%	PACE trial; dose equivalence
27	[52]	Küpper et al., 2008	Germany	RCT, DB, PC	2 arms	17	3,440 m	LLS ≥ 3	THEO/PL	THEO 350 mg SR BD	THEO 4/9; PL 1/8	THEO 44%; PL 13%	Small sample; ventilatory stimulus
28	[53]	Talbot et al., 2011	UK/Peru	RCT, DB, PC	2 arms	24	4,340 m (Peru)	LLS ≥ 3	IV iron/PL (saline)	Fe-sucrose 200 mg IV	Fe 7/12; PL 4/12	Fe 58%; PL 33%	Oxford hypoxia group
29	[54]	Hohenhaus et al., 1994	Germany	RCT, DB, PC	2 arms	27	4,559 m	LLS ≥ 3	NIF/PL	NIF 20 mg SR TID	NIF 5/14; PL 5/13	NIF 36%; PL 38%	No AMS prevention effect
30	[55]	Luks et al., 2007	USA	RCT, DB, XO	2 arms	16	Hypobaric chamber (FiO₂ 12.4%)	LLS ≥ 3	MTK/PL	MTK 10 mg/d × 4 d	MTK 0/8; PL 0/8	0%; 0%	Chamber study; leukotriene blockade
31	[56]	Heo et al., 2014	South Korea	RCT, DB, PC	2 arms	39	3,840 m (Korea)	LLS ≥ 3	EPO/PL	EPO 300 IU/kg SC	EPO 14/20; PL 5/19	EPO 70%; PL 26%	EPO pre-treatment; unusual finding
32	[57]	Basnyat et al., 2003	Nepal	RCT, DB, PC	2 arms	155	4,928 m	LLS ≥ 3	ACZ 125 mg/PL	ACZ 125 mg BD	ACZ 65/74; PL 61/81	ACZ 88%; PL 75%	Low-dose efficacy trial
33	[58]	Moraga et al., 2007	Chile	RCT, DB, PC	2 arms	24	3,696 m	LLS ≥ 3	ACZ/PL	ACZ 250 mg BD	ACZ 8/12; PL 5/12	ACZ 67%; PL 42%	Chilean altitude study
34	[59]	Furian et al., 2022	Switzerland	RCT, DB, PC	2 arms	345	3,100 m	LLS 2018 ≥ 3	ACZ/PL	ACZ 250 mg BD	ACZ 137/175; PL 116/170	ACZ 78%; PL 68%	Older adults (>60 y); large RCT
35	[60]	Carlsten et al., 2004	Bolivia	RCT, DB, PC	4 arms	63	3,630 m (La Paz)	LLS ≥ 3	ACZ 125/250/375 mg/PL	3 dose levels BD	ACZ 18/21; PL 9/11	86%; 82%	Dose-response in tourists
36	[61]	Gertsch et al., 2010	USA	RCT, DB, PC	3 arms	162	3,810-4,300 m	LLS ≥ 3	ACZ/IBU/PL	ACZ 250 mg BD; IBU 600 mg TID	ACZ 79/97; PL 47/65	ACZ 81%; PL 72%	HEAT trial
37	[62]	Yang et al., 2019	China	RCT, DB, PC	2 arms	37	3,700-4,500 m	LLS ≥ 3	TMZ/PL	TMZ 20 mg TID	TMZ 12/19; PL 11/18	TMZ 63%; PL 61%	Elderly tourists; metabolic modulator
38	[63]	Salvi et al., 2013	Italy/Argentina	RCT, DB, PC	2 arms	39	3,260-5,079 m	LLS ≥ 3	ACZ/PL	ACZ 250 mg BD	ACZ 13/19; PL 6/20	ACZ 68%; PL 30%	Cardiac function focus
39	[64]	Li et al., 2016	China	RCT, DB, PC	3 arms	124	3,800 m	LLS ≥ 3	BUD inh/DEX/PL	BUD 800 µg/d; DEX 8 mg/d	BUD 32/42; DEX 27/39; PL 17/43	BUD 76%; DEX 69%; PL 40%	Inhaled vs. systemic steroid
40	[65]	Bärtsch et al., 1991	Switzerland	RCT, DB, PC	2 arms	21	4,559 m	HAPE/AMS criteria	NIF/PL	NIF 20 mg SR TID	NIF 9/10; PL 2/11	NIF 90%; PL 18%	HAPE-susceptible subjects; landmark
41	[66]	Maggiorini et al., 2006	Switzerland	RCT, DB, PC	3 arms	29	4,559 m	LLS ≥ 3	TAD/DEX/PL	TAD 10 mg BD; DEX 8 mg/d	TAD 3/10; DEX 7/10; PL 1/9	TAD 30%; DEX 70%; PL 11%	HAPE-susceptible subjects
42	[67]	Basnyat et al., 2008	Nepal	RCT, DB, PC	2 arms	364	4,928 m	LLS ≥ 3	ACZ/PL	ACZ 250 mg BD	ACZ 168/187; PL 138/177	ACZ 90%; PL 78%	Nepali porters; largest porter trial
43	[68]	Bates et al., 2011	UK/Nepal	RCT, DB, PC	2 arms	62	5,200 m	LLS ≥ 3	SLD/PL	SLD 50 mg TID	SLD 5/20; PL 9/42	SLD 25%; PL 21%	Pulmonary hypertension focus
44	[69]	Burtscher et al., 2019	Austria	RCT, DB, PC	2 arms	15	Normobaric hypoxia (4,000 m sim)	LLS ≥ 3	ACZ/PL	ACZ 250 mg BD	ACZ 6/8; PL 2/7	ACZ 75%; PL 29%	Simulated altitude chamber
45	[70]	Mutschler et al., 2023	Switzerland	RCT, DB, PC	2 arms	95	3,454 m	LLS 2018 ≥ 3	ACZ/PL	ACZ 250 mg BD	ACZ 46/57; PL 24/38	ACZ 81%; PL 63%	Pediatric study
46	[71]	Wang et al., 2018	China	RCT, DB, PC	3 arms	121	3,700 m (rapid ascent)	LLS ≥ 3	BUD inh/SAL inh/PL	BUD 800 µg/d; SAL 200 µg BD	BUD 30/31; SAL 56/60; PL 27/30	BUD 97%; SAL 93%; PL 90%	Military personnel; rapid ascent
